# EBP1 promotes the malignant biological behaviors of kidney renal clear cell carcinoma through activation of p38/HIF-1α signaling pathway

**DOI:** 10.1186/s12935-024-03442-x

**Published:** 2024-07-24

**Authors:** Huan Meng, Shuxia Cao, Shengri Tian, Jiaqi Huo, Xiangdan Li, Dongyuan Xu, Lan Liu

**Affiliations:** 1 Center of Morphological Experiment, Yanbian University, Yanji, 133002 Jilin China; 2 Key Laboratory of Cellular Function and Pharmacology of Jilin Province, Yanbian University, Yanji, 133002 Jilin China; 3 Department of Pathology, Yanbian University Hospital, Yanji, 133000 Jilin China

**Keywords:** Kidney renal clear cell carcinoma, ERBB3 binding protein, p38/HIF-1α signaling pathway

## Abstract

**Background:**

Kidney Renal Clear Cell Carcinoma (KIRC) is a common malignant tumor of the urinary system, and its incidence is increasing. ERBB3 binding protein (EBP1) is upregulated in various cancers. However, the connection between EBP1 and KIRC has not been reported.

**Methods:**

The expression of EBP1 in normal kidney tissue and KIRC tissue was analyzed through database and tissue microarray. EBP1 was knocked down in KIRC cell lines, and its impact on KIRC proliferation was assessed through CCK-8, soft agar assay, and flow cytometry. Scratch and transwell assays were used to evaluate the influence of EBP1 on KIRC invasion and migration. Nude mice tumor experiment were conducted to examine the effect of EBP1 on tumor tissue. Database analysis explored potential pathways involving EBP1, and validation was performed through Western blot experiments and p38 inhibitor.

**Results:**

EBP1 is upregulated in KIRC and significantly correlates with clinical staging, pathological grading, and lymph node metastasis in patients. The mechanism research showed that knocking down EBP1 inhibited KIRC proliferation, invasion, and migration and inhibited p38 phosphorylation and the expression of hypoxia-inducible factor-1α (HIF-1α) in KIRC. p-38 inhibitor (SB203580) inhibits p38 phosphorylation and HIF-1α expression and suppresses cell viability in a concentration-dependent manner, but has no effect on EBP1 expression. HEK 293T cells overexpressing EBP1 showed increased expression of phosphorylated p38 and HIF-1α and enhanced cell viability, however, SB203580 inhibited this effect of EBP1.

**Conclusion:**

EBP1 may promote the occurrence and development of KIRC by regulating the expression of p38/HIF-1α signaling pathway.

**Graphical Abstract:**

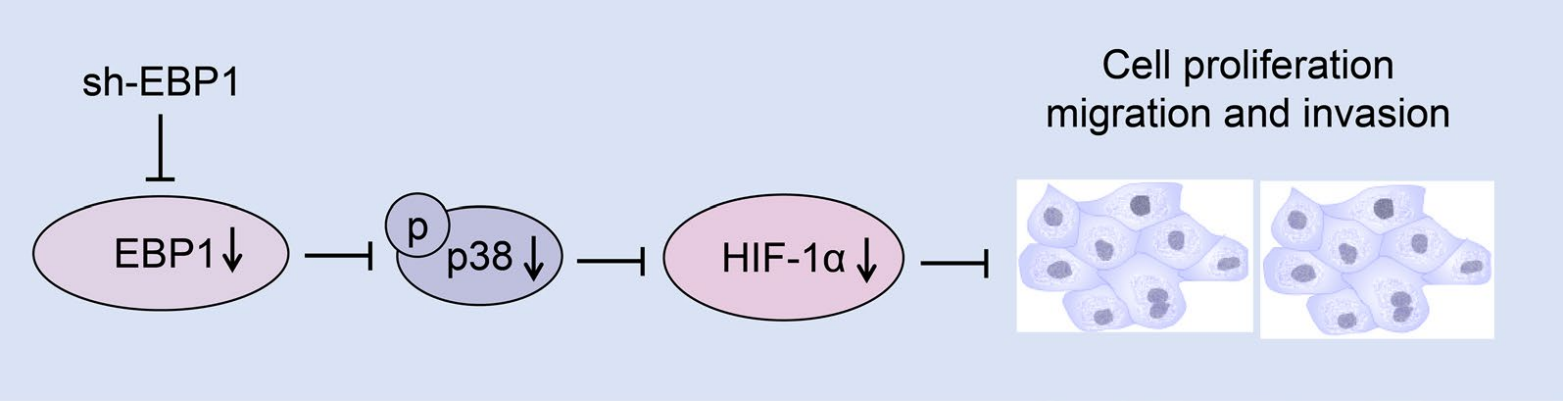

## Introduction

Kidney Clear Cell Carcinoma (KIRC) is a common malignant tumor of the urinary system, accounting for approximately 4.2% of all adult malignancies [1]. The incidence of KIRC has been steadily increasing in recent years. Early-stage KIRC is often asymptomatic and challenging to detect, posing significant difficulties in treatment when hematuria symptoms manifest, indicating tumor invasion behind the renal pelvis. Despite efforts to enhance early diagnosis through the identification of biomarkers, effective predictive factors for KIRC remain lacking.

proliferation-associated 2G4, also known as ERBB3 binding protein 1 (EBP1), plays a crucial role in cellular development, proliferation, and apoptosis [2]. EBP1 has two subtypes, p42 and p48, with opposing functions [3]. While p42 exhibits tumor growth inhibitory effects in the cytoplasm [2], suppressing the growth of tumors such as prostate cancer, non-small cell lung cancer, and breast cancer [3]. p48 found in the cell nucleus and cytoplasm, it was found that EBP1 can directly interact with Bcl-2 mRNA and thus promote Bcl-2 expression [4]. It has been implicated in promoting cancer, for example, in hepatocellular carcinoma [5] and melanoma [6]. However, the role of EBP1 in KIRC is poorly documented. EBP1 p48, the predominant form of EBP1 [6], deserves more attention for its potential oncogenic effects.

Studies have shown that knocking down EBP1 significantly affects the growth rate of mice compared to wild-type mice, causing alterations in proteins associated with the mitogen-activated protein kinase (MAPK) signaling pathway [7]. p38, a member of the MAPK protein family, plays an irreplaceable role in various aspects of cell proliferation, differentiation, death, embryonic development, and different aspects of tumor progression [8]. The activation of p38 is associated with the progression of various human diseases, such as its activation following renal ischemia/hypoxia injury [9]. Hypoxia-inducible factors (HIFs) are primary regulatory elements responding to hypoxia, with the activation of HIF-1α closely associated with poor prognosis in cancer [10]. It has been identified that HIF-1α is involved in various processes of renal tumor occurrence and development, including initiation, progression, and therapeutic outcomes [11]. Gregg L Semenza [12] discovered that HIF-1α can promote the growth of KIRC by enhancing the Warburg effect. Simultaneously, studies indicate that inhibiting MAPK in KIRC can disrupt the expression of the HIF-1α gene, emphasizing the crucial role of MAPK in HIF-1α expression [13]. Therefore, we speculate that EBP1 may potentially facilitate the progression of KIRC by modulating the expression of p38 and HIF-1α.

Building upon the current research status and our preliminary groundwork, we utilized bioinformatics, immunohistochemistry, and other methods to screen and identify the expression of EBP1 in KIRC. Additionally, we analyzed the correlation between the expression of EBP1 and clinical pathological parameters. Based on these findings, we employed molecular biology techniques in cultured cells and nude mice experiments to further explore the role and molecular mechanisms of EBP1 in the growth, proliferation, invasion, and metastasis of KIRC. This research provides a theoretical basis for understanding the occurrence and development of KIRC and identifying new therapeutic targets.

## Methods and materials

### KIRC tissue microarrays

The KIRC tissue microarrays used in the experiment were purchased from Shanghai Outdo Biotech Co., Ltd. The microarrays included 90 cases of normal kidney tissue and 90 cases of KIRC tissue.

### Cell culture

786-O, 769-P and Caki-1, HEK 293T cells were purchased from the National Collection of Authenticated Cell Cultures. 786-O, 769-P cells were cultured in RPMI 1640 medium, Caki-1, HEK 293T cells were cultured in DMEM medium (Gibco, New York, USA) containing 10% FBS and 1% penicillin-streptomycin at 37 °C in a 5% CO2 incubator. Cell lines were tested monthly for mycoplasma and validated via STR testing.

### CCK-8 assay

Cells treated with various interventions (such as lentiviral transfection or inhibitor treatment) were seeded at 1 × 10^3^ cells per well in a 96-well plate. At 1, 2, 3, 4, 5, and 6 days, 10 µL of CCK-8 solution (Beyotime, Shanghai, China) was added, and the absorbance was measured at 450 nm after 2 h of incubation.

### Soft agar colony formation experiment

A total of 1 × 10^5^ cells were mixed with a 0.7% upper agar layer and then added to 6 cm culture dishes containing a 1.4% lower agar layer. After 2 weeks of incubation at 37 °C in a constant temperature incubator, cell colony formation was observed under a microscope.

### Cell cycle detection

Cells were fixed overnight in precooled absolute ethanol, resuspended, and centrifuged at 400 *g* for 5 min. After discarding the ethanol, the cells were incubated with propidium iodide (PI) for 40 min at room temperature in the dark. Cell cycle analysis was performed using the FACS Calibre flow cytometer (BD Biosciences).

### Scratch assay

1 × 10^5^ cells were seeded in a 6-well plate until reaching confluence. A 1 mL pipette tip was used to scratch the cells, and the scratch width was recorded at 0 h and 48 h.

### Transwell assay

Cells were starved in serum-free RPMI 1640 medium for 24 h, and then 2 × 10^5^ cells were seeded in the upper chamber. The lower chamber was filled with RPMI 1640 or DMEM medium containing 10% FBS and incubated at 37 °C for 24 h. After fixation with 4% paraformaldehyde for 15 min, cells were stained with Giemsa overnight. Photographs were taken under a microscope.

### Subcutaneous tumor experiment in Nude mice

Nude mice required for the experiment were obtained from the Yanbian University Animal Center, and the experimental protocol was compliance with Directive 2010/63/EU in Europe and approved by the Yanbian University Animal Ethics Committee. Ten 4-week-old female nude mice were randomly divided into two groups. Cells (5 × 10^6^ cells, 786-O sh-NC, sh-EBP1) suspended in matrix gel were injected subcutaneously into the right upper limb of the mice. Mice were kept for 30 days, euthanized, and tumor tissues were collected, fixed in formalin, and embedded in paraffin.

### H&E staining

Tumor tissues were sectioned into 4 μm slices, deparaffinized in xylene, dehydrated in a graded ethanol series, stained with H&E following the instructions of the H&E staining kit, differentiated in 1% hydrochloric acid alcohol, hydrated in a graded ethanol series, and finally, sealed with neutral resin. Photographs were taken under a microscope.

### Immunohistochemistry (IHC)

Slides were baked overnight at 56 °C, and antigen retrieval was performed with sodium citrate buffer (OriGene, Wuxi, China). Primary antibodies, including EBP1 (NT) (ABE43, Millipore, USA), was incubated overnight at 4 °C. After washing with PBS, slides were incubated with the corresponding secondary antibodies at room temperature for 30 min. Immunohistochemistry was performed following the instructions of the two-step immunohistochemistry kit (ZSGB-BIO, Beijing, China). After DAB staining, photographs were taken under a microscope.

### Western blot

Whole-cell lysis was performed using RIPA cell/tissue lysis buffer (Solarbio, Beijing, China) and concentration identification of extracted proteins using BCA standard protein kit (Thermo Fisher, USA). Subsequently, proteins were separated by SDS-PAGE, transferred onto PVDF membranes, and incubated overnight at 4 °C with primary antibodies: EBP1(NT), p38 (sc-81,621, Santa Cruz, USA, ), p-p38(Tyr182)(sc-166,182, Santa Cruz, USA), HIF-1α (ab179483, Abcam, UK, ), and GAPDH (sc-47,724, Santa Cruz, USA). After a 1.5-hour incubation with corresponding secondary antibodies at room temperature, the membranes were developed.

### Lentivirus transfection experiment

786-O and 769-P cell lines were transfected with a shRNA lentiviral vector encoding EBP1 and a control shRNA lentiviral vector (SyngenTech, Beijing China). The sequence for EBP1 was CCGGCCACCAGCATTTCGGTAAATACTCGAGTATTTACCGAAATGCTGGTGGTTTTTTTG, and the control sequence was CCGGCCTGGTCGTGACCAAGTATAACTCGAGTTATACTTGGTCACAGGTTTTTG. 1 × 10^5^ cells were seeded in a 6-well plate, infected with the lentivirus for 24 h, and then selected with 1 g/mL puromycin to obtain successfully transfected cells. EBP1 overexpression lentiviral vector and control vector were purchased from Beijing SyngenTech Co., Ltd, China (ref number: pHS-AVC-LY124, pHS-AVC-ZQ328). Transfection efficiency was assessed by western blot.

### Statistical analysis

ImageJ was used for image analysis, and GraphPad Prism 10.0 and SPSS 28.0 were employed for statistical analysis of experimental data. The t-test was used for comparisons between two groups, and comparisons between multiple groups were analyzed by one-way ANOVA. Data are presented as mean ± SD, and statistical significance was considered when *P* < 0.05.

## Results

### EBP1 is highly expressed in KIRC

**Fig. 1:**
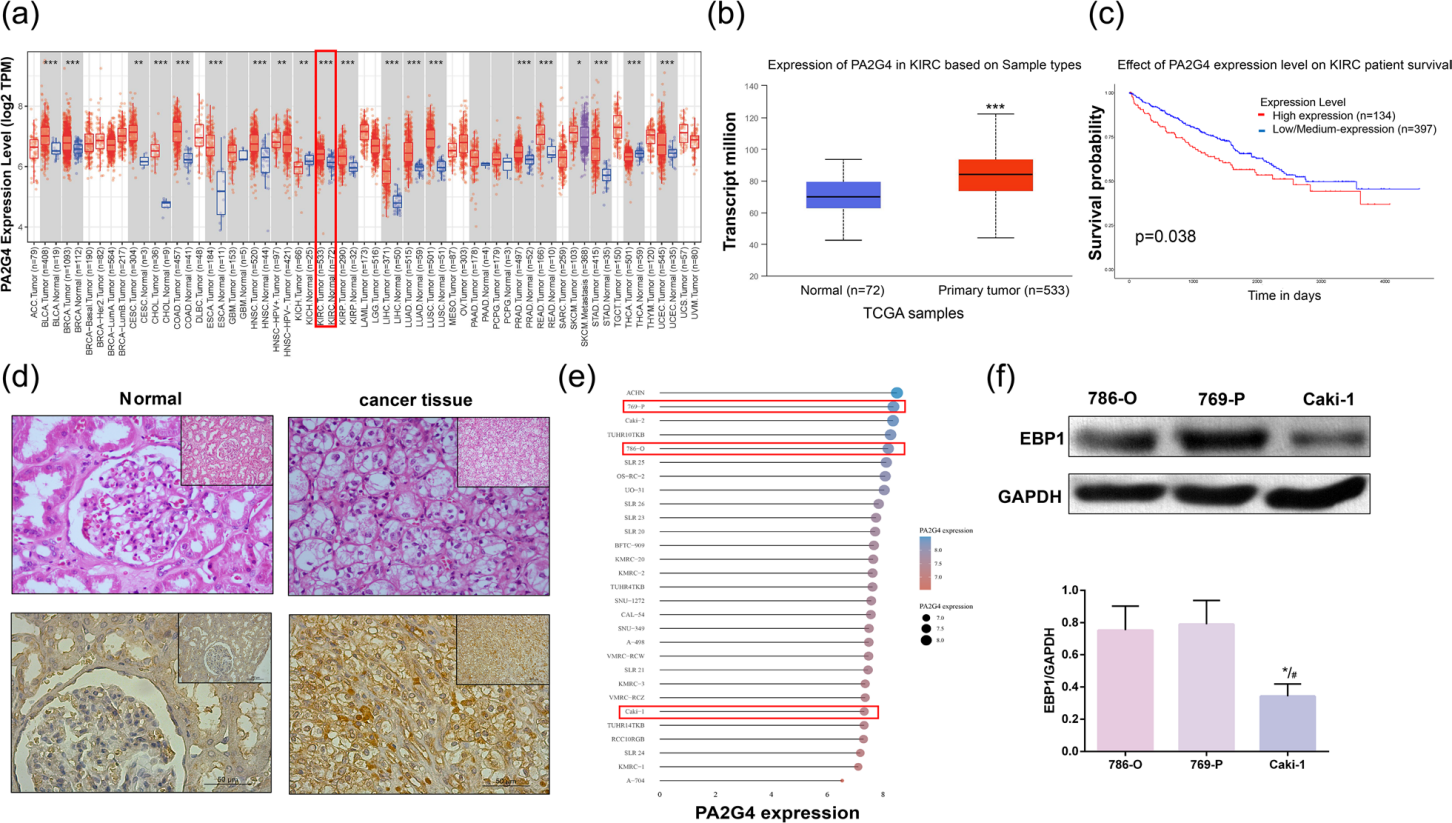
EBP1 is highly expressed in KIRC. **a** EBP1 is expressed in pan-cancer, *P < 0.05, **P < 0.01, ***P < 0.001 VS. normal. **b** EBP1 expression in normal tissue and KIRC, ***P < 0.001 VS. normal. **c** Effect of EBP1 expression on prognostic survival of KIRC patients. **d** H&E staining, IHC for normal tissue and KIRC tissue alterations. **e** Expression of EBP1 in different cells. **f** western blot analysis of EBP1 expression levels in 786-O, 769-P and Caki-1, *P < 0.05 VS. 786-O, ^#^*P* < 0.05 VS. 769-P, *n* = 3

The Cancer Genome Atlas (TCGA) database (https://www.cancer.gov/ccg/research/genome-sequencing/tcga) revealed a significant increase in EBP1 expression in KIRC tissues compared to normal tissues (Fig. 1a, b). Survival curves demonstrated that patients with high EBP1 expression had significantly lower survival rates than those with low EBP1 expression (Fig. 1c), indicating a clear correlation between EBP1 expression and patient survival. Next, we examined the expression of EBP1 in tissue microarrays by IHC, which showed a high positivity rate of 45.6% in KIRC tissues compared to 16.7% in normal kidney tissues (Table 1). A representative picture is shown in Fig. 1d. EBP1 was strongly positively expressed in the nucleus and cytoplasm of KIRC tissues, whereas in normal tissues EBP1 was expressed only in small amounts in the cytoplasm. H&E staining showed loss of normal glomerular structure and transparent cytoplasm. To analyze the expression levels of EBP1 in KIRC lines, we obtained a gene expression matrix of KIRC lines from the CCLE database (https://sites.broadinstitute.org/ccle/) (Fig. 1e). We selected specific cell lines of interest and validated the intracellular expression of EBP1 through western blot, revealing that EBP1 expression in 786-O and 769-P was significantly higher than in A498 (Fig. 1f). Thus, 786-O and 769-P were chosen for subsequent experiments.

**Table 1:** EBP1 expression in KIRC and normal tissues

Type	Number		EBP1				Positivity rate	*p-*value
			(-)	(+)	(++)	(+++)		
KIRC		90	49	10	18	13	45.6%	*P*<0.05
Normal		90	75	9	6	0	16.7%	

### Clinical pathological significance of EBP1 expression in KIRC patients

To further investigate the clinical pathological significance of EBP1 expression in KIRC patients, we conducted IHC experiments to assess EBP1 expression in clinical tissues from 90 cases. Table 2 provides clinical pathological information for the patients. We observed a significant correlation between EBP1 expression and clinical staging, pathological grading, and lymph node metastasis in KIRC patients (Table 3).

**Table 2:** Clinical data of the study population

Clinical information	Classification	Number of cases
Age(years)	<65	58
	≥ 65	32
Gender	Male	59
	Female	31
Region	Left	46
	Right	44
Magnitude	Diameter<3 cm	9
	Diameter ≥ 3 cm	81
Pathological grading	I-III	42
	IV	48
Clinical staging	I	55
	II-IV	35
Lymphatic node metastasis	Yes	6
	No	84

**Table 3:** Relationship between EBP1 expression and clinicopathological features in KIRC

Clinicopathological features		N	EBP1				Positivity rate(%)	*p-*value
			(-)	(+)	(++)	(+++)	(+) ~ +++	
Age (Years)	<65	58	34	5	10	9	41.4%	*P*>0.05
	≥ 65	32	17	5	7	3	46.9%	
Gender	Female	59	32	8	11	8	45.8%	*P*>0.05
	Male	31	18	2	7	4	41.9%	
Region	Left	46	28	5	7	6	39.1%	*P*>0.05
	Right	44	25	4	9	6	43.2%	
Magnitude(cm)	Diameter < 3	9	7	0	0	2	22.2%	*P*>0.05
	Diameter ≥ 3	81	45	9	17	10	44.4%	
Pathological grading	I-III	42	32	2	8	0	23.8%	*P<*0.05
	IV	48	17	8	10	13	64.6%	
Clinical staging	I	55	43	3	7	2	21.8%	*P*<0.05
	II-IV	35	6	7	11	11	82.9%	
Lymphatic node metastasis	Yes	6	0	2	3	1	100%	*P*<0.05
	No	84	49	8	15	12	41.7%	

### Knockdown of EBP1 suppresses KIRC cell proliferation

**Fig. 2:**
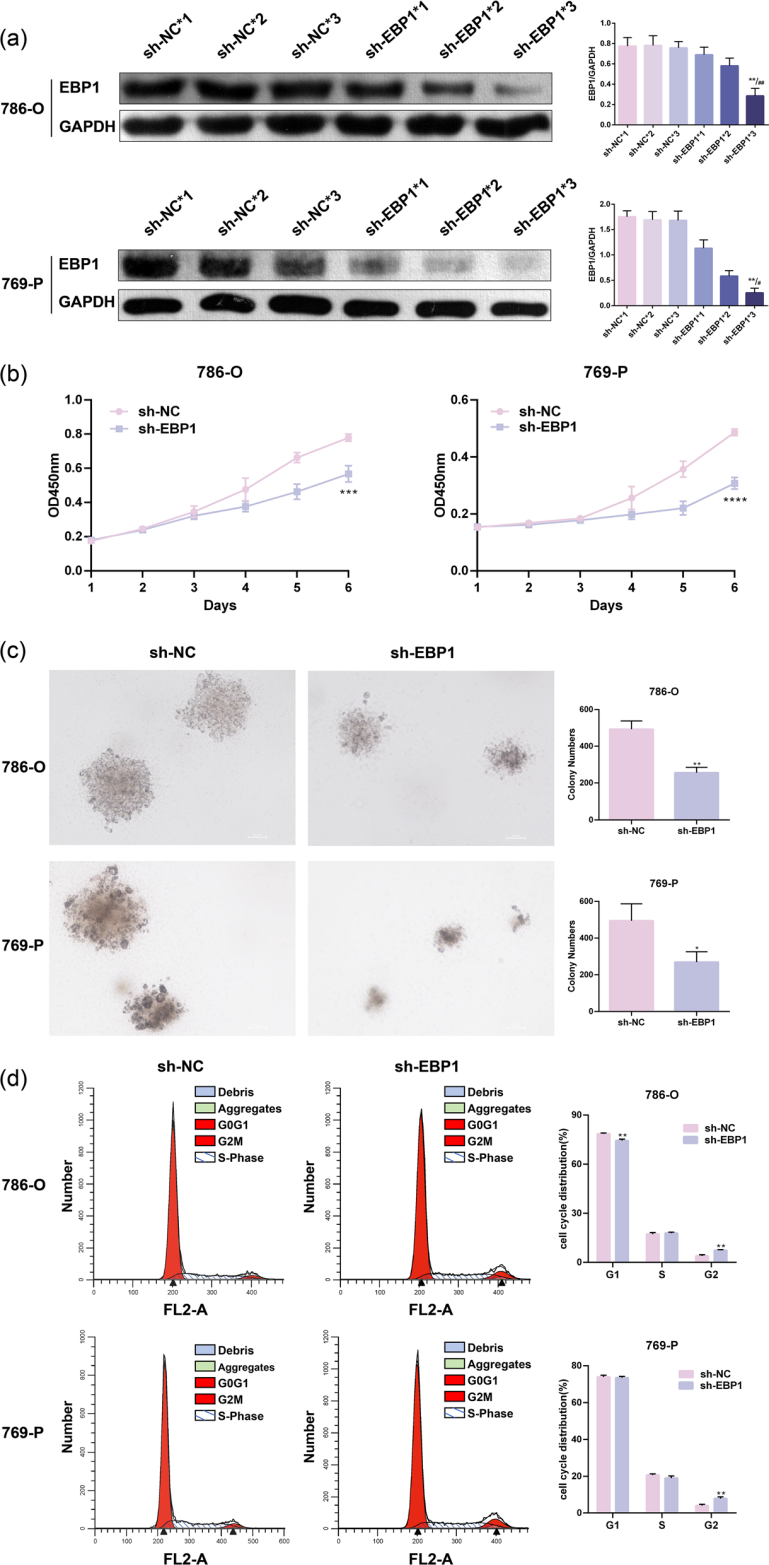
Knockdown of EBP1 suppresses KIRC cell proliferation. **a** Western blot to detect the efficiency of lentiviral transfection, **P < 0.01 VS. sh-EBP1*1, ^#^*P* < 0.05, ^##^*P* < 0.01 VS. sh-EBP1*2. **b** CCK-8 assay to assess the impact of EBP1 knockdown on cell viability. **c** Colony formation assay to evaluate the effect of EBP1 knockdown on cell proliferation. **d** Flow cytometry analysis to investigate the impact of EBP1 knockdown on the cell cycle. *P < 0.05, **P < 0.01, ***P < 0.001, ****P < 0.0001 VS. sh-NC, *n* = 3

The Western blot results showed EBP1 is highly expressed in 786-O and 769-P cells (Fig. 1f). To investigate the biological function of EBP1 in KIRC cells, stable knockdown of EBP1 was achieved in 786-O and 769-P cells using shRNA. The transfection efficiency was confirmed by Western blot, and sh-EBP1*3 showed the most effective knockdown (Fig. 2a). Consequently, sh-NC*3 and sh-EBP1*3 were chosen for subsequent experiments. CCK-8 results revealed a significantly reduced growth rate in cells with EBP1 knockdown compared to control cells (Fig. 2b). Colony formation assay results demonstrated a marked decrease in colony-forming ability in KIRC cells with EBP1 knockdown (Fig. 2c). To further understand the reasons behind the inhibitory effect on cell proliferation, cell cycle analysis was performed, revealing a noticeable G2/M phase arrest in KIRC cells after EBP1 knockdown (Fig. 2d). In conclusion, knocking down EBP1 induced G2/M phase arrest, suppressing the proliferation of KIRC cells.

### Knockdown of EBP1 suppresses KIRC cell migration

**Fig. 3:**
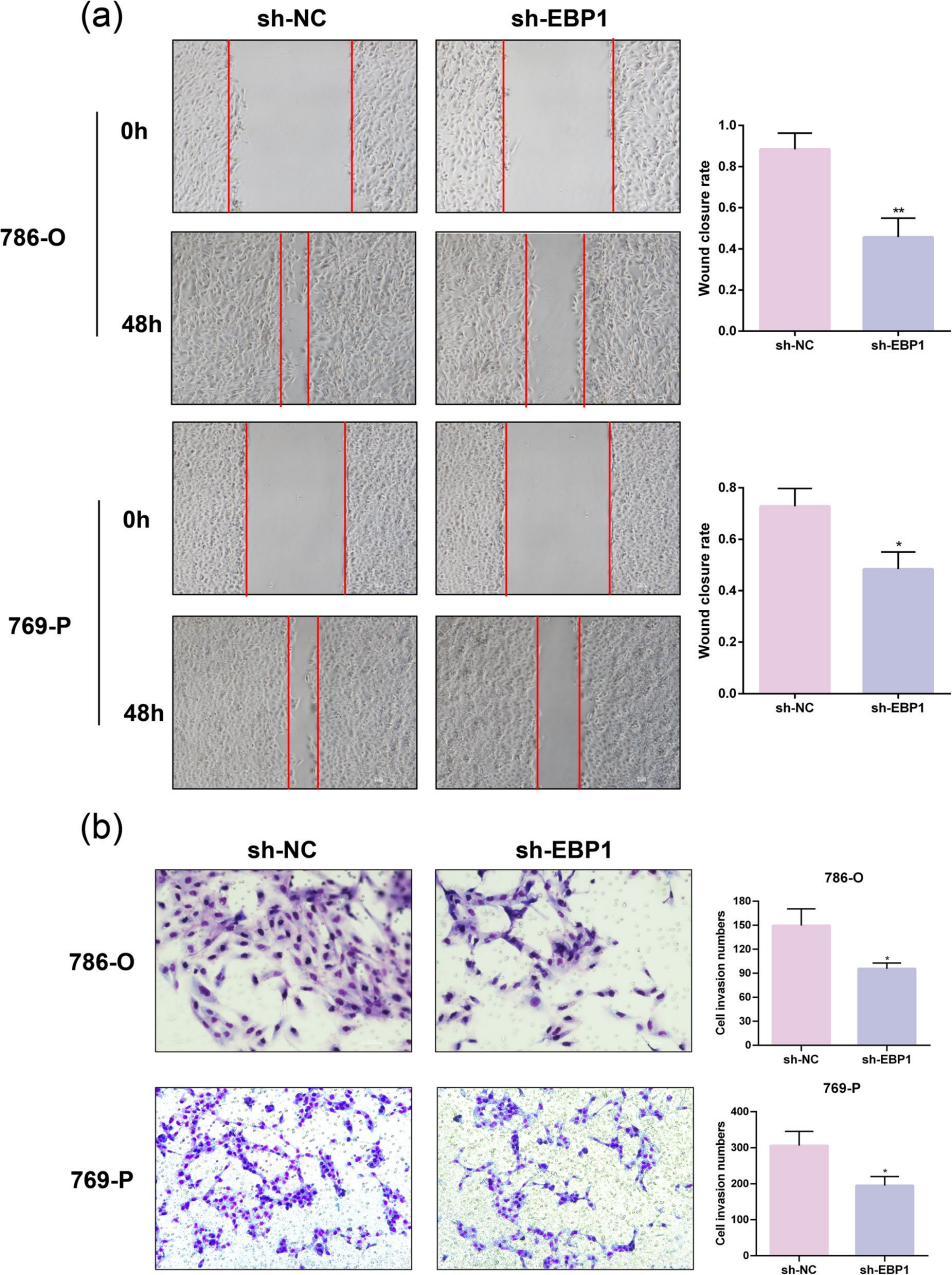
Knockdown of EBP1 suppresses KIRC cell migration. **a** Scratch assay to detect the effect of knockdown of EBP1 on the migratory capacity of KIRC cells. **b** Transwell assay to detect the effect of knockdown of EBP1 on the invasive ability of KIRC cells. *P < 0.05, **P < 0.01 VS. sh-NC, *n* = 3

Next, we examined the impact of EBP1 knockdown on the migration ability of KIRC cells. Scratch assay results demonstrated a significant reduction in cell migration ability after EBP1 knockdown compared to the sh-NC group (Fig. 3a). The results of the Transwell experiment indicate that downregulating EBP1 significantly reduced the invasive capacity of KIRC cells (Fig. 3b). The above experimental findings suggest that knocking down EBP1 can inhibit the migratory ability of KIRC cells.

### Knockdown of EBP1 inhibits the growth of mouse xenograft tumors

**Fig. 4:**
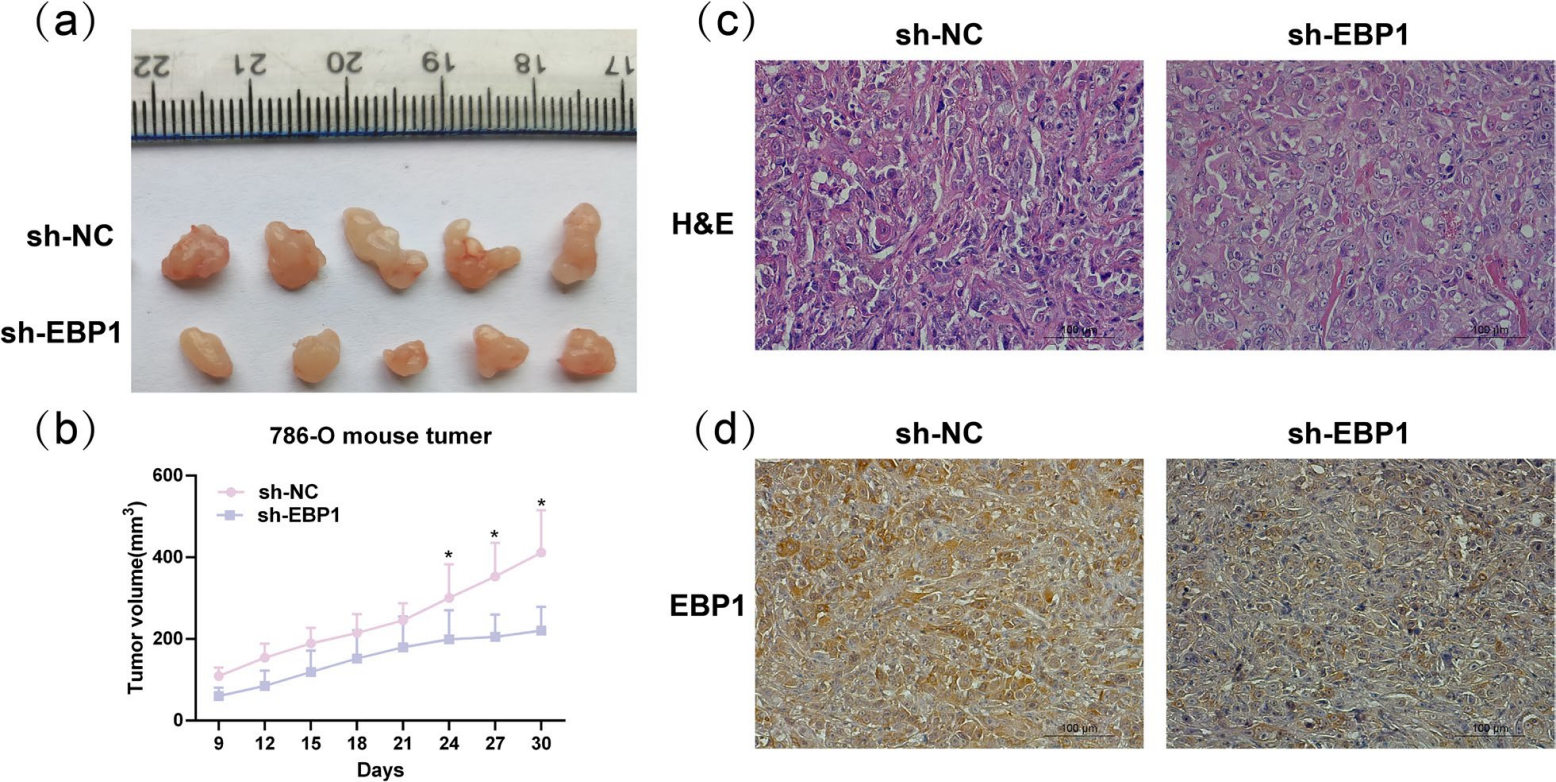
Knockdown of EBP1 inhibits the growth of mouse xenograft tumors. **a** The impact of EBP1 knockdown on tumor growth. **b** Tumor growth within 30 days. **c** H&E staining of tumor tissues in the sh-NC group and sh-EBP1 group. **d** IHC detection of EBP1 expression in tumor tissues. *P < 0.05 vs. sh-EBP1, *n* = 5

To validate the impact of knockdown of EBP1 on tumor growth in vivo, we injected 786-O cells with knocked down EBP1 or untreated cells subcutaneously into mice. The experimental results demonstrate a significant reduction in tumor volume after knocking down EBP1 compared to the control group (Fig. 4a, b). Figure 4c illustrates the H&E staining of tumors in the sh-NC and sh-EBP1 groups. IHC results that EBP1 expression was significantly reduced in tumor tissues in the sh-EBP1 group compared to the sh-NC group (Fig. 4d). These experimental findings suggest that knocking down EBP1 significantly inhibits the growth of KIRC in vivo.

### Knockdown of EBP1 suppresses the p38/HIF-1α signaling pathway

**Fig. 5:**
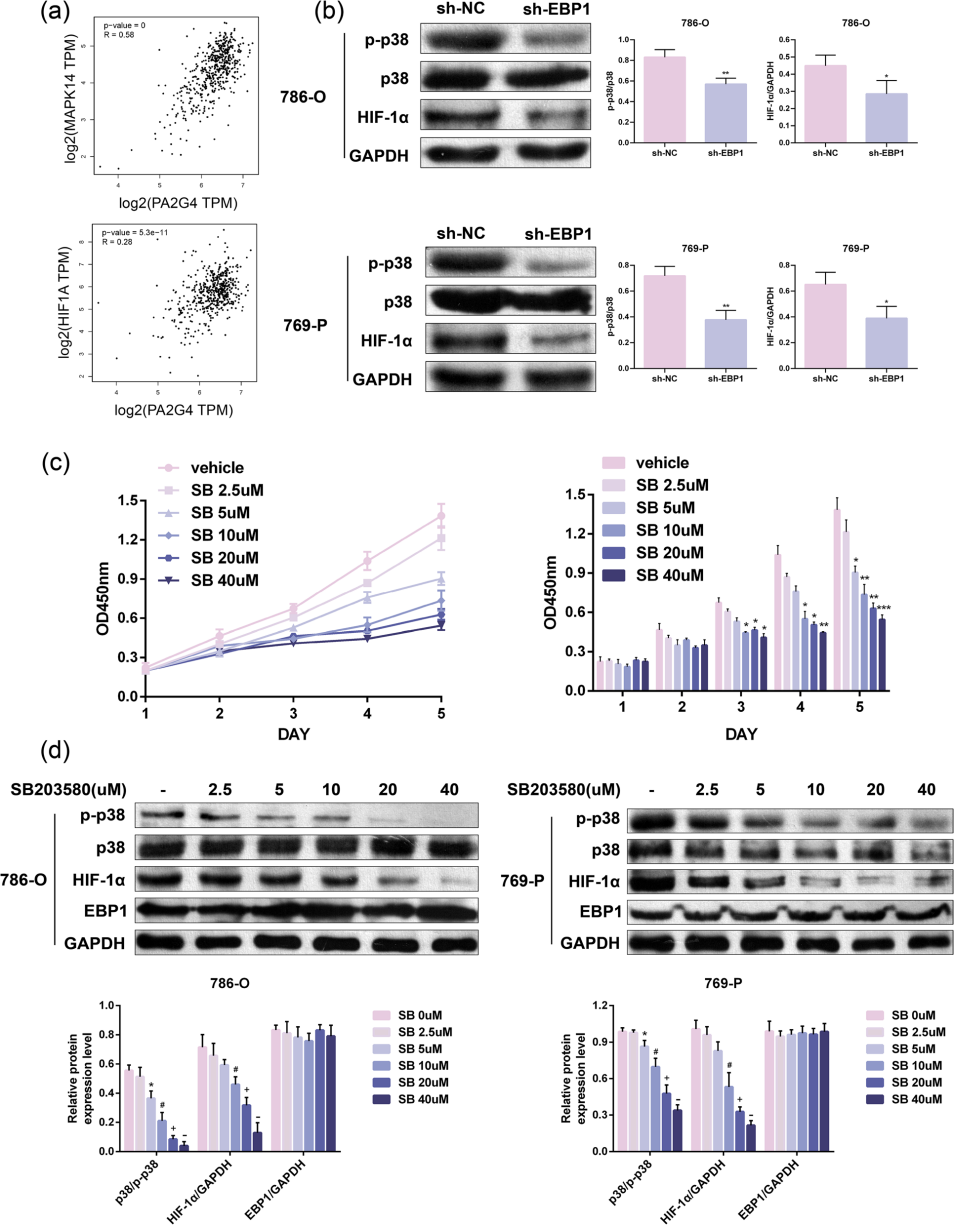
Knockdown of EBP1 p48 suppresses the p38/HIF-1α signaling pathway. **a** GSEA database analysis of the relationship between EBP1 and p38, HIF-1α. **b** Western blot to assess the impact of knocking down EBP1 on p38 phosphorylation and HIF-1α expression, *P < 0.05, **P < 0.01 VS. sh-NC. **c** Impact of p38 inhibitor SB203580 on the viability of 786-O cells, *P < 0.05, **P < 0.01, ***P < 0.01 VS. vehicle, *n* = 4. **d** Effect of p38 inhibitor SB203580 on HIF-1α and EBP1, *P < 0.05 VS SB2.5µM, ^#^*P* < 0.05 VS. SB5µM, ^+^*P* < 0.05 VS. SB10µM, ^−^*P* < 0.05 VS. SB20µM. Data presented as mean ± SD, *n* = 3

To investigate the mechanism of EBP1, we explored the relationship between EBP1 and p38 as well as HIF-1α using the Gene set enrichment analysis (GSEA) database. The results indicated a positive correlation between EBP1 and p38, HIF-1α (Fig. 5a). Western blot experiments revealed that knocking down EBP1 significantly inhibited the phosphorylation of p38 and the expression of HIF-1α in KIRC cells (Fig. 5b). Subsequently, we treated 786-O cells with SB203580 (a p38 inhibitor, referred to as SB), we found that 786-O cell viability gradually decreased with increasing SB concentration (Fig. 5c). Western blot results showed that p38 phosphorylation and HIF-1α expression gradually decreased with increasing SB concentration, with no impact on EBP1 expression (Fig. 5d). These experiments collectively indicate that knocking down EBP1 can suppress the p38/HIF-1α signaling pathway.

#### EBP1 acts through the p38/HIF-1α signaling pathway

**Fig. 6:**
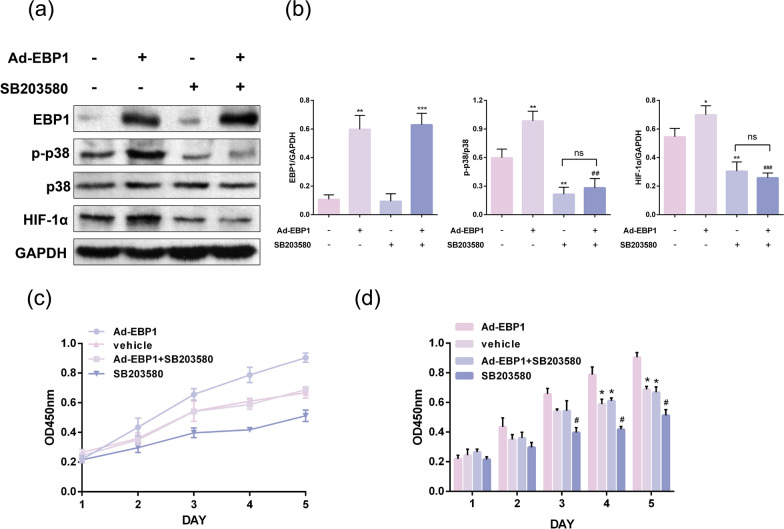
EBP1 acts through the p38/HIF-1α signaling pathway. **a** western blot assay to detect the effect of overexpression of EBP1 or SB203580 treatment on p38 and HIF-1α. **b** Quantitative analysis of proteins, *P < 0.05, **P < 0.01 VS. Ad-EBP1(-) + SB203580(-), ^##^*P* < 0.01, ^###^*P* < 0.001 VS. Ad-EBP1(+) + SB203580(-), *n* = 3. **c** CCK-8 assay to detect the effect of overexpression of EBP1 or SB203580 treatment on HEK 293T cell viability, *P < 0.05 VS. Ad-EBP1, ^#^*P* < 0.05 VS. vehicle/Ad-EBP1 + SB203580, *n* = 4

We next overexpressed EBP1 in HEK 293T cells and then gave SB treatment. Increased expression of phosphorylated p38, HIF-1α after overexpression of EBP1 alone compared to blank group (Fig. 6a, b). Decreased expression of phosphorylated p38, HIF-1α and no change in EBP1 expression after SB treatment alone (Fig. 6a, b). Whereas overexpression of EBP1 and SB co-treatment resulted in SB inhibiting this effect of EBP1 (Fig. 6a, b). HIF-1α expression was reduced compared to the overexpression of EBP1 group alone, but almost unchanged compared to the SB treatment group alone (Fig. 6a, b). CCK-8 results showed that HEK 293T cell viability was significantly enhanced after overexpression of EBP1 alone compared to the blank control group (Fig. 6c, d). while cell viability was significantly reduced after treatment with SB alone (Fig. 6c, d). Overexpression of EBP1 co-treated with SB resulted in no change in cell viability compared to controls (Fig. 6c, d). The above experimental results indicate that SB inhibits the action of EBP1, suggesting that EBP1 may act through the p38/HIF-1α signaling pathway.

## Discussion

While the incidence of KIRC among malignant tumors is not high, its occurrence is inconspicuous due to the concealed location of the kidneys, making early detection challenging for patients. The insensitivity of KIRC to radiotherapy and chemotherapy further complicates late-stage treatment [14]. Therefore, identifying a biomarker to enhance early diagnosis of KIRC is crucial. Initially, our database analysis revealed upregulation of EBP1 expression in KIRC. Subsequent experiments, involving the knockdown of EBP1 in KIRC cell lines, demonstrated a significant inhibition of proliferation, invasion, and migration capabilities, suggesting a correlation between EBP1 and the progression of KIRC.

EBP1 is a proliferation-associated protein [15], and its two subtypes, p42 and p48, seem to play different roles in various diseases [16]. Current research indicates that EBP1 p48, by binding to the WD domain of FBXW, can promote tumor tissue growth [17]. Our previous studies found that EBP1 p48 can promote the progression of malignant melanoma by activating the Wnt/β-catenin signaling pathway [6]. In this study, we observed high expression of EBP1 in KIRC, and knocking down EBP1 resulted in a significant decrease in cell viability and proliferation, indicating a promoting role of EBP1 in KIRC progression, consistent with previous research. However, in another urological tumor, prostatic cancer, EBP1 expression was found to decrease, inhibiting the growth of prostatic cancer cells upon EBP1 activation [18], the results of the experiment are contrary to the results of our experiment. Given the divergent results and considering previous studies showing that overexpression of EBP1 p42 can inhibit the proliferation of breast cancer cells [19], we speculate that the differential roles of the two EBP1 subunits may contribute to their varied effects in different tumors.

In vivo and in vitro experiments concurrently demonstrate that knocking down EBP1 can inhibit the progression of KIRC. To further elucidate the molecular mechanism underlying this phenomenon, we used a database to predict potential downstream molecules affected by EBP1. Database results indicate a linear relationship between EBP1 and p38 as well as HIF-1α. Previous studies have shown that the activation of p38 MAPK and HIF-1α promotes the metastasis of breast cancer cells [20]. The p38 signaling pathway plays a crucial role in the progression of cancer. Activated p38 can regulate the production of matrix metalloproteinases (MMPs) [21], which are associated with tumor migration. In this study, we observed that knocking down EBP1 inhibited the phosphorylation level of p38. Additionally, to further confirm the impact of p38 on KIRC cell proliferation, we treated 786-O and 769-P cells with the p38 inhibitor SB203580. The results indicated that the p38 inhibitor dose-dependently inhibited KIRC cell viability, consistent with previous research. Researchers are currently exploring the development of a new anticancer drug targeting p38, with clinical trials already underway [22].

HIF-1α serves as a substrate for various kinases, including p38, and is a major regulatory mechanism in tumor tissues responding to hypoxia. Tumor tissues undergo local hypoxia due to unrestricted proliferation, leading to increased reliance on glycolysis as the primary energy source. Various enzymes involved in glycolytic pathways are regulated by HIF-1α. HIF-1α binds to various enzymes and leads to modification of metabolic pathways such as angiogenesis in cancer cells, this encourages the growth of tumors tissue [23]. In our study, we found a gradual reduction in HIF-1α expression with increasing doses of the p38 inhibitor. Tyrosine kinase inhibitors (TKIs) are common drugs used in the clinical treatment of KIRC. For example, imatinib and dasatinib [24]. However, KIRC is resistant to TKIs for a number of reasons [25]. When tissue hypoxia increases the expression of HIF-1α, which targets multiple genes downstream, including vascular endothelial growth factor (VEGF), epidermal growth factor (EGF), and others [11]. These factors also increase KIRC resistance to TKIs by promoting angiogenesis and tumour growth [26]. An inhibitor of HIF has recently been approved for the treatment of renal cell carcinoma [27]. In this experiment we found that the expression of HIF-1α was reduced after knocking down EBP1, so we speculated that knocking down EBP1 might enhance the therapeutic effect of TKIs. As for the effect of EBP1 on TKI we will continue to validate and collect further clinically relevant data to demonstrate the effect of EBP1 on TKI in subsequent experiments.

Notably, the use of the p38 inhibitor did not alter the expression of EBP1, indicating that EBP1 is downstream of p38 and HIF-1α. To further elucidate the mechanism of EBP1 action, we overexpressed EBP1 in HEK 293T cells and showed that phosphorylated p38 and HIF-1α expression increased after overexpression of EBP1. However, after administration of SB, the expression level of HIF-1α was unchanged even after overexpression of EBP1. The results of the present study suggest that EBP1 promotes the progression of KIRC by activating the p38/HIF-1α signaling pathway. These findings provide new ideas for early detection and treatment of KIRC.

## Conclusions

p38 and HIF-1α are downstream signaling molecules of EBP1. EBP1 promotes KIRC cell proliferation, migration and invasion by inhibiting the p38/HIF-1α signaling pathway. Inhibition of EBP1 protein expression interferes with the proliferation, invasion and metastatic ability of KIRC tumor cells.

## Data Availability

No datasets were generated or analysed during the current study.

## Abbreviations

KIRC: Kidney Clear Cell Carcinoma
EBP1: ERBB3 binding protein
HIF-1α: Hypoxia-inducible factor-1α
MAPK: Mitogen-activated protein kinase
IHC: Immunohistochemistry
TCGA: The Cancer Genome Atlas
TKI: Tyrosine kinase inhibitors
